# 3-Methylglutaconyl-Coenzyme-A Hydratase Deficiency and the Development of Dilated Cardiomyopathy

**DOI:** 10.14740/cr359w

**Published:** 2014-10-06

**Authors:** Craig D. Spergel, Mariya Milko, Christopher Edwards, Jeff P. Steinhoff

**Affiliations:** a Largo Medical Center, 201 14th Street Southwest, Largo, FL 33770, USA

**Keywords:** 3-Methylglutaconyl-coenzyme-A hydratase deficiency, 3-Methylglutaconic aciduria type I, 3-MGA, Aciduria, Cardiomyopathy, Dilated, Heart failure, Organic aciduria, MGCA1, 3-MGA type I

## Abstract

A 25-year-old Canadian male with a history of 3-methylglutaconyl-coenzyme-A hydratase deficiency, also known as 3-methylglutaconic aciduria type I, a very rare inborn error of metabolism, presented with respiratory distress, nausea, vomiting and signs of multisystem organ failure due to a suspected underlying infectious process. An electrocardiogram revealed bilateral atrial enlargement and an elevated brain natriuretic peptide on the initial laboratory studies, which prompted a more thorough cardiac workup. The transthoracic echocardiogram revealed a dilated cardiomyopathy with severe systolic dysfunction. The deficient enzyme present in this patient is involved in the pathway of leucine catabolism and is particularly important in various tissues for energy production and sterol synthesis. The dilated cardiomyopathy in this patient possibly had a variety of potential mechanisms including: a mitochondrial myopathy due to the deficiency of this enzyme leading to a defect in energy production inside cardiac myocytes; or a direct toxicity from 3-methylglutaconic acid (3-MGA) and its toxic metabolites; or a cardiac dysfunction due to a variety of other potential mechanisms. In conclusion, this patient’s clinical presentation suggested that 3-methylglutaconyl-CoA hydratase deficiency could cause a severe dilated cardiomyopathy and heart failure.

## Introduction

3-Methylglutaconyl-CoA hydratase deficiency (OMIM #250950) is an extremely rare autosomal recessive disorder characterized by the urinary excretion of abnormal quantities of 3-methylglutaconic acid (3-MGA) and the metabolites 3-methylglutaric and 3-hydroxyisovaleric acids. As per records from the “Online Mendelian Inheritance in Man” database, fewer than 20 cases have been reported, all with varying degrees of symptom severity. This deficiency impairs the metabolism of leucine during mitochondrial energy production causing a buildup of acids that are excreted in the urine. In addition, this inefficient mitochondrial energy production is believed to profoundly affect all tissues that have a higher metabolic rate (and therefore higher energy requirement), including neurons, adipocytes, hepatocytes, skeletal and cardiac myocytes.

## Case Report

A 25-year-old white male from Newfoundland, Canada, with a past medical history significant for 3-methylglutaconyl-CoA hydratase deficiency, confirmed with a record of 3-MGA in urine organic acid analysis and skin biopsy fibroblast analysis, developmental learning delays, motor deficits, childhood seizures and cyanotic breath-holding spells since approximately 1 year of age, was brought to the emergency department (ED) by his father. The patient had two healthy living siblings and one deceased brother born with severe congenital abnormalities who died 24 h post-partum. According to his father, the patient experienced 5 days of progressive weakness, nausea, vomiting, and suprapubic pain. The night prior to admission, the patient felt dyspneic and developed a productive cough with white frothy sputum. Upon presentation to the ED, the patient was hypotensive, tachycardic, encephalopathic, and in respiratory failure with an O_2_ saturation around 50% on room air. The initial arterial blood gas with 100% oxygen on resuscitation revealed a partially compensated metabolic acidosis with a pH of 7.035, pCO_2_ of 11.9 mm Hg, pO_2_ of 236.3 mm Hg, HCO_3_ of 2.6 mEq/L and a base excess of -26 with an anion gap of 33 mEq/L. Subsequent labs revealed a lactic acid of 22 mmol/L and a procalcitonin of 13.57 ng/mL. The patient demonstrated signs of septic shock and end organ involvement with a blood glucose of 16 mg/dL, troponin of 0.09 ng/mL, elevated ammonia level at 104, elevated bilirubin, AST, ALT, hypovolemic hyponatremia, hyperkalemia, and acute kidney injury with a BUN of 29 mg/dL and creatinine of 2.6 mg/dL. The patient was intubated in the ED and placed on mechanical ventilation. A triple lumen central venous catheter was placed and the patient was given fluid resuscitation followed by a norepinephrine drip for refractory hypotension. The patient was started on both empiric vancomycin and piperacillin/tazobactam and was transferred to the intensive care unit after stabilization.

**Figure 1:**
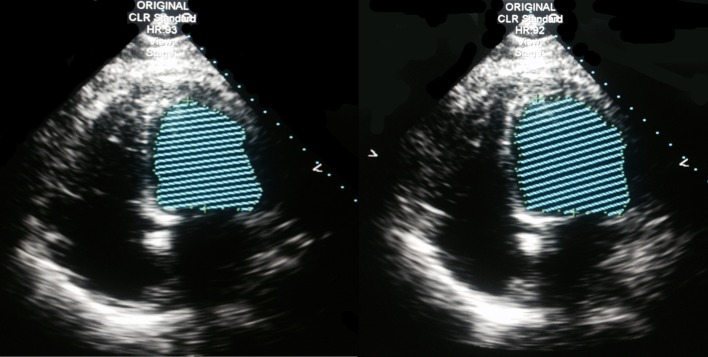
2D echocardiogram, four-chamber view, Simpson method of ejection fraction estimation. Estimated ejection fraction: 15-20%, four-chamber dilatation.

The initial CT imaging of chest, abdomen, and pelvis revealed small bilateral pleural effusions with atelectasis and/or infiltrate in the lower lungs, a hypodense gallbladder with sludge, and fatty infiltration of liver. Blood and sputum cultures were unremarkable as there was no growth reported. It was not completely clear what infectious organism or process was the initial cause of the patient’s clinical picture, although pneumonia or an intra-abdominal process was the most suspicious for the cause. It was less likely that the patient’s symptoms were related directly to acute congestive heart failure and developing metabolic acidosis, given the elevated procalcitonin level. The initial EKG showed sinus tachycardia and biatrial enlargement. The brain natriuretic peptide was above 5,000 pg/mL and an echocardiogram was ordered. The 2D echocardiogram revealed a dilated left ventricle with global, severely reduced systolic function with an estimated ejection fraction of 15-20%. There were no areas of regional variation. The right ventricular systolic pressure was estimated at 55 mm Hg. The inferior vena cava was dilated with blunted respirophasic changes (Fig. 1). The cardiology service was subsequently consulted.

Given the findings and patient’s history of 3-methylglutaconyl-CoA hydratase deficiency, there was a suspicion that the patient’s dilated cardiomyopathy could potentially be a mitochondrial myopathy secondary to his genetic metabolic disorder. Various other types of cardiomyopathies such as cardiomyopathy of sepsis, Tako Tsubo’s cardiomyopathy, or direct toxicity from organic acids were also considerations. Given his young age and low suspicion for acute coronary syndrome, cardiac angiography was not performed. The patient was carefully weaned off vasopressors and was started on beta-blockers. Various extended runs of supraventricular tachycardia complicated patient’s hospital course and required up-titration of beta-blockers. When the renal failure had resolved, ACE-inhibitors were also started. An external ICD (Lifevest) was recommended for the primary prevention of sudden cardiac death. It was also recommended that the patient undergo a repeat 2D echocardiogram in 9 months to re-evaluate for recovery of contractile function and to determine his eligibility for an AICD to decrease the risk of sudden cardiac death from cardiac dysrythmia. The patient was subsequently transferred to a tertiary care center in Canada for further medical management.

## Discussion

3-Methylglutaconyl-CoA hydratase deficiency (MGCA1) (OMIM #250950), one of many organic aciduria diseases, is type I of five subtypes known to cause 3-methylglutaconic aciduria. MGCA1 is caused by a homozygous or compound heterozygous mutation in the AUH gene (OMIM *600529) on chromosome 9q22, which encodes 3-methylglutaconyl-CoA hydratase (3-MGH). This autosomal recessive disorder is extremely rare with a prevalence of less than 1 in 1,000,000. 3-MGH is the enzyme responsible for the fifth step of leucine catabolism, specifically catalyzing the conversion of 3-methylglutaconyl-CoA to HMG-CoA, which is then broken down in the mitochondria during the process of energy production. A deficiency of this enzyme leads to a buildup of 3-MGA, which is excreted in the urine along with its derivatives, 3-methylglutaric acid (3-MG) and 3-hydroxyisovaleric acid (3-HIVA) [1]. The breakdown of the essential amino acid leucine has been found to occur primarily in the liver, adipose and muscle tissue; however, there has been evidence that branched-chain amino acids (BCAAs), which include leucine, are catabolized in non-hepatic tissues, including cardiac myocytes, neurons, and kidney tissue as well [2]. Leucine is used extensively in the formation of various sterols, including cholesterol precursors. In murine studies, 3-MGH has been shown to be highly expressed in kidney, skeletal muscle, heart, liver and spleen, and was shown to be located in the mitochondria [3]. The clinical features of 3-methylglutaconic aciduria type I are quite variable, but typically include speech and psychomotor delays, dystonia, spasms, weakness of the arms and legs, and the characteristic elevated levels of acid in the urine, blood and tissues.

In times of increased metabolic demand, such as acute febrile illness and severe infection, there is increased need for energy to maintain homeostasis. In these times, the catabolism of amino acids aids in energy production is vital for cellular processes. This catabolic stress causes an endogenous proteolysis that releases amino acids for energy production [4]. In patients with 3-MGA type1, the amino acid leucine cannot be completely broken down and thus the organic acid 3-MGA and its metabolites accumulate causing a metabolic acidosis. This process, as in other organic acidurias, is typically accompanied by serum hypoglycemia due to increased intracellular glucose breakdown in response to increased intracellular energy demand. Serum hyperammonemia also occurs due to secondary inhibition of the urea cycle by 3-MGA [5]. In organic acidemias, the accumulated toxic organic acids, and their esters in the mitochondria, inhibit carbamylphosphate synthetase I (CPS-I), which is the first enzyme found in the urea cycle, in turn causing hyperammonemia. These states are often episodic, usually in periods of increased metabolic demand when organic acid levels are elevated in patients with organic acidurias [6]. As stated above, the above patient was markedly hypoglycemic and markedly with an extreme elevation of serum hyperammonemic, both of which resolved with appropriate treatment.

In the case of our patient, we present that the enzyme deficiency contributed to the development of a dilated non-ischemic cardiomyopathy with severe systolic dysfunction. Cardiomyopathy is not previously known to be associated with 3-methylglutaconic aciduria type I; however, it is commonly associated with type II 3-methylglutaconic aciduria (Barth syndrome) (OMIM #302060), which is caused by a mutation in the tafazzin gene which codes for a protein crucial for maintaining proper levels of cardiolipin in the membrane of mitochondria and classically presents with heart defects associated with familial dilated cardiomyopathy. Cardiomyopathy has also been associated with 3-MGA type V (OMIM #610198), characterized by dilated cardiomyopathy and ataxia. There have also been various reports of cardiomyopathy with type 3-MGA type IV [7-12]. Myocardial cells are rich in mitochondria and rely heavily on energy production to maintain proper function for efficient cardiac output. To our knowledge, this is the first reported case of a dilated cardiomyopathy associated with a patient diagnosed with type I 3-methylglutaconic aciduria. There are various potential pathophysiologic mechanisms that could explain our patient’s resultant dilated cardiomyopathy with severe systolic dysfunction.

### Potential pathophysiology mechanisms of cardiomyopathy in studied patient

#### Direct 3-MGA and toxic metabolite cytotoxicity

BCAAs such as leucine are normally metabolized to acetyl-CoA and succinyl-CoA, which are then consumed in the mitochondria through the tri-carboxylic acid (TCA) cycle for the production of nicotinamide adenine dinucleotide (NADP) for respiration [13]. Leucine has also been shown to have potent signaling activity in cells to promote cellular metabolism. While BCAAs such as leucine are essential for normal cellular function, excessive amounts of free BCAA or their catabolic products have shown to be cytotoxic. It has been shown that increased metabolic demand due to sepsis causes an increase in amino acid catabolism [4]. In our patient, this would result in increased production of the organic acid 3-MGA and its metabolites, 3-MG and 3-HIVA due to his enzyme deficiency impairing leucine catabolism. These specific organic acids have been shown to cause an oxidative stress on tissues that could result in cellular dysfunction and death. The “accumulation of toxic metabolites may give rise to slow-onset excitotoxicity with cellular dysfunction and eventually cell death” [1]. It has been previously demonstrated that 3-MGA exhibits a toxic effect on the cerebral cortex in rats [14]. There is also evidence that 3-MGA and 3-HIVA accumulation is both neurotoxic and hepatotoxic and can cause brain damage [15, 16]. It has been demonstrated that the enzyme 3-MGH, which is deficient in patients with 3-MGA type I, is highly expressed in heart tissue [3]. It is reasonable to assume that during this state of extreme metabolic demand, buildup of toxic metabolites in heart tissue could cause significant damage, although to our knowledge, there are no significant studies regarding this issue as of yet. There has been some evidence that catabolic intermediates of leucine, and other BCAAs, may trigger cardiac dysfunction although, as of this date, the direct impact of leucine and its catabolic intermediate products directly on mitochondrial performance and contractile function in the heart has not been studied [17].

#### Dysfunction of mitochondrial-targeted 2C-type ser/thr protein phosphatase (PP2Cm)-mediated BCAA catabolism

There have been studies performed on PP2Cm, which is involved in BCAA metabolism in cardiac myocytes. In a study on zebrafish embryos, when PP2Cm was inactivated and BCAA metabolism compromised, there was induced apoptosis, a dose-dependent loss in cardiac contractility and premature death of cardiac myocytes. These studies have provided evidence that BCAA catabolism is essential for normal cellular function in cardiac tissue; therefore, PP2Cm-mediated catabolism of BCAAs may be a significant contributor in the pathophysiology of cardiac diseases [18]. It is plausible that impairment of leucine catabolism, an important BCAA, can potentially have an effect on the normal cellular function of cardiac tissue, especially in states of metabolic stress. It has been demonstrated that PP2Cm expression is more prevalent in vital organs such as brain, heart and diaphragm muscles of adult mice compared to other tissues [18]. This study implies that BCAA catabolism is quite important to vital organs. The above study demonstrated that murine cardiomyocytes under physiologic stress demonstrate diminished PP2Cm expression, which in turn leads to increased levels of intracellular free BCAAs [18]. In times of metabolic stress, such as demonstrated in this patient, the inability to properly catabolize leucine may have played a role in the inability to maintain appropriate cardiac muscle function and even diaphragmatic muscle function. Furthermore, the increased free BCAA leucine would be unable to be appropriately catabolized, leading to further increases in 3-MGA and its toxic metabolites, potentially causing cardiac dysfunction from inability to meet metabolic demands and inability for the cardiac tissue to appropriately respond to physiologic stress. There would also be a potential direct toxicity effect from the organic acid and its metabolites as stated above [19].

#### Cardiomyopathy of sepsis

It is also plausible that the stress of septic shock and multi-system organ dysfunction, due to his suspected bacterial pneumonia (given the elevated procalcitonin level), caused a stress-induced cardiomyopathy, for which the patient would be at higher risk due to his metabolic disorder and subsequent inability to meet metabolic demands. There is not a confirmed mechanism for the etiology of sepsis-induced cardiomyopathy, although there has been much research into the pathogenesis of this condition including: sepsis-induced alterations in myocardial flow; alterations in microcirculation; sepsis-induced increases in myocardial depressive substances such as tumor necrosis factor, interleukin and complement anaphylatoxin as well as various other factors; metabolic stress and oxygen debt; autonomic dysregulation; disruption of ATP-dependent calcium transport; a decrease in the density of calcium L-channels or a decreased sensitivity of myofilaments to calcium causing depressed contractility and impairment of systolic function; excessive stimulation of b-adrenergic receptors causing myocardial damage via intracellular calcium overload and cell necrosis; and mitochrondial dysfunction from endotoxin-induced mitochrondial DNA damage causing decreased oxygen consumption. Myocardial function typically improves or resolves within 7 - 10 days in patients with this condition [20].

#### Tako Tsubo cardiomyopathy

A cardiomyopathy is associated with times of extreme emotional or physical stress identified by acute apical ballooning of the left ventricle causing reversible left ventricular dysfunction. The current exact etiology of this condition is yet unknown. In order to diagnose this disorder, other well-known pathologies such as coronary artery disease, cerebrovascular etiology, pheochromocytoma, viruses, tachycardia-induced cardiomyopathy and idiopathic myocarditis must be excluded. Confirmation of this disorder typically requires repeat echocardiography documenting significant improvement or normalization of left ventricular ejection fraction by 1 year since the event, although the majority of patients are female and middle-aged [21].

#### Lactic acidosis and severe acidemia-induced cardiomyopathy

Lactic acidosis can occur when significant tissue hypoxia or the body’s ability to buffer by generating appropriate amounts of bicarbonate is overwhelmed. There have been various studies examining the effects of lactic acidosis and the effects of pH changes on hemodynamics, cardiac muscle contractility, and left ventricular function [22, 23]. In our patient, severe lactic acidosis was present on admission, which also may have contributed to his significant cardiac dysfunction.

### Conclusions

Due to the rarity of this disease, it is difficult to wholly determine if this patient’s development of dilated cardiomyopathy falls within the spectrum of this disorder, or if it is secondary to a different etiology, but the possible association is important to consider. As discussed above, there are various differential etiologies of the systolic dysfunction seen in this patient, including acute cardiomyopathy of sepsis and Tako Tsubo cardiomyopathy, both of which require documentation of the resolution or improvement of the systolic function, which cannot be determined until a repeat surface echocardiogram is performed in December of 2014. It is also possible that the patient developed his cardiomyopathy due to severe acidemia or from a direct toxic effect of increased 3-MGA and its metabolites on cardiac tissue. In addition, it is also possible that in the patient’s time of high metabolic stress, enzymatic pathways, such as PP2Cm and/or other pathways not discussed in this paper, including chronic induction of cardiac mTor from increased leucine concentration in the stressed heart from impaired leucine catabolism resulting in suppression of cardioprotective authophagy, caused the acute cardiac dysfunction [19, 24, 25]. Due to the possibility that patients with this disorder may develop a cardiomyopathy secondary to their disease and may be at higher risk for cardiac dysfunction during times of metabolic stress, further clinical study, including but not limited to ECG and echocardiography, as well as laboratory testing of the cytotoxicity of 3-MGA and its metabolites directly on heart tissue, may be warranted. In our patient, there was no documentation of a 2D echocardiogram performed during early childhood and a repeat serum organic acid analysis was not readily available.

Therefore, in the treatment of patients with 3-MGCA1, cardiac involvement should be considered, especially in times of significantly increased metabolic demand such as septic shock. Current treatment for 3-MGCA1 is limited and mostly symptomatic. Current recommendations are that patients should maintain a leucine restricted diet [1] and in some cases, supplementation with L-carnitine may result in additional therapeutic benefits [26, 27]. Although more conclusive studies must yet be performed, a high index of suspicion for cardiac involvement may lead to early diagnosis and treatment to help prevent progression to severe heart failure and improve the quality of life and overall patient outcomes.

## Author Note

All authors can be reached at Largo Medical Center.
